# Combined wet milling and heat treatment in water vapor for producing amorphous to crystalline ultrafine Li_1.3_Al_0.3_Ti_1.7_(PO_4_)_3_ solid electrolyte particles†

**DOI:** 10.1039/d1ra02039k

**Published:** 2021-04-21

**Authors:** Takahiro Kozawa

**Affiliations:** Joining and Welding Research Institute, Osaka University 11-1 Mihogaoka Ibaraki Osaka 567-0047 Japan t-kozawa@jwri.osaka-u.ac.jp

## Abstract

Bulk-type all-solid-state batteries (ASSBs) consisting of composite electrodes of homogeneously mixed fine particles of both active materials and solid electrolytes (SEs) exhibit a high safety, high energy density, and long cycle life. SE nanoparticles are required for the construction of ion-conducting pathways as a response to the particle size reduction of active materials; however, simple and low-cost milling processes for producing nanoparticles cause a collapse in the crystal structure and eventually amorphization, decreasing the conductivity. This study develops a heat treatment process in water vapor for the low-temperature crystallization of ultrafine SE amorphous particles and the size control of crystalline nanoparticles. An ultrafine (approximately 5 nm) amorphous powder of Li_1.3_Al_0.3_Ti_1.7_(PO_4_)_3_ (LATP), as a typical oxide-type SE, is produced *via* wet planetary ball milling in ethanol. The water vapor induces a rearrangement of the crystal framework in LATP and accelerates crystallization at a lower temperature than that in air. Further, since particle growth is also promoted by water vapor, depending on the heating temperature and time, this heat treatment process can be also applied to the size control of crystalline LATP nanoparticles. A combination of the wet planetary ball milling and heat treatment in water vapor will accelerate the practical application of bulk-type ASSBs.

## Abstract 2

The preparation of ultrafine particles through combined wet planetary ball milling and heat treatment in water vapor contributes to the fabrication of extensive composite electrodes with solid electrolytes for bulk-type all-solid-state batteries.

## Graphical abstract

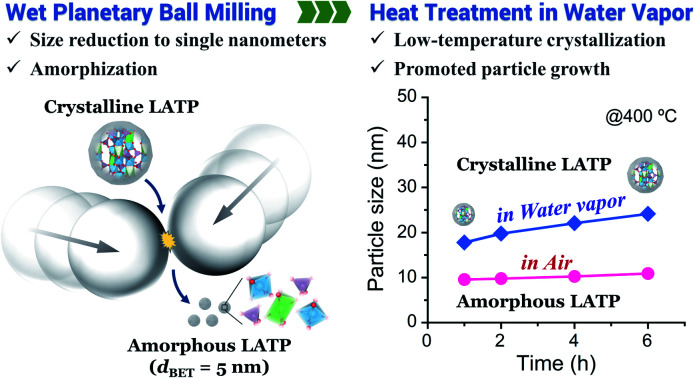

## Introduction

All-solid-state batteries (ASSBs) have attracted significant attention as next-generation batteries because they can solve the safety issue that results from the organic liquid electrolytes used in current Li-ion batteries.^1–3^ Traditional electrolytes possess safety risks due to the flammability of organic solvents and HF formation that attacks the active materials of the electrodes.^4,5^ Solid electrolytes (SEs), on the other hand, offer high stabilities against temperature, air, and moisture; hence, replacing liquid electrolytes with SEs contributes to the high safety and cycle life of Li-ion batteries. Further, their stable potential window beyond 5 V allows the application of high-voltage cathodes (*e.g.*, Li-rich-layered Ni/Mn/Co compounds, LiNi_0.5_Mn_1.5_O_4_, LiCoPO_4_, *etc.*) that induce the oxidative decomposition of organic solvents during the charging process.^6–8^ This advantage leads to a significant increase in the energy density of ASSBs.

Electrodes for bulk-type ASSBs consist of a composite of homogeneously mixed fine particles of both active materials and SEs. An increase in the mass loading of the active material, (*i.e.*, a decrease in the SE ratio) results in a high gravimetric energy density. However, simply adjusting the weight ratio alone decreases battery capacity.^9^ This is because the particle size of SEs and their size ratio with active materials are important for constructing ion-conducting pathways in the composite electrode.^10,11^ Meanwhile, the use of smaller SE particles permits an increase in the mass loading of active materials due to the relative increase in the particle size ratio. According to Shi *et al.*,^11^ the use of composites with large cathode particles (∼12 μm) and small SE particles (∼1.5 μm) allowed the realization of liquid-cell-level cathode volume loading and capacities. While a decrease in SE particle size is required to increase the particle size ratio, cathode particles, especially in olivine-type materials with high thermal stabilities, tend to be smaller in size in terms of their low ionic conductivities.^12–14^ Accordingly, it will be necessary to prepare ultrafine SE particles on the nanometer scale in the future.

Milling processes are widely accepted as low-cost particle size reduction processes; the preparation of nanoscale ultrafine particles can be achieved by using high-energy mills. Although milling processes for a long time are a simple way to produce fine SE particles, they cause a collapse in the crystal structure and eventual amorphization. Certain oxide-type SEs need to be crystallized because of their low ionic conductivity in the amorphous state.^15–18^ Further, their increased surface area results in the strong adsorption of milling solvents at the particle surface after milling. Heat treatment at high temperatures for crystallization or removal of the adsorbed materials causes Li evaporation and a side reaction with the active materials within the composites to form a reactive layer.^19,20^ Understanding the surface state of milled products, alongside the size and crystallinity, is important for applying the milling process to the production of ultrafine SE particles. Moreover, after milling, a heating process that promotes crystallization at low temperatures is desirable to prevent Li evaporation. Controlling the size of crystalline SE nanoparticles through low-temperature heat treatment is more challenging.

In this study, ultrafine amorphous particles (approximately 5 nm) of Li_1.3_Al_0.3_Ti_1.7_(PO_4_)_3_ (LATP), which is a typical oxide-type SE (oxidation potential, ∼4.2 V *vs.* Li/Li^+^), are prepared *via* a simple wet milling process of commercially available LATP powder for 4 h. The crystallinity decreases with decreasing particle size and finally transforms into the amorphous phase. Surface and thermal analyses reveal that the organic species originating from the ethanol, used as the milling solvent, are adsorbed on the resulting particles. Herein, the introduction of water vapor into heat treatment is proposed for the low-temperature crystallization of amorphous LATP. The prepared amorphous LATP crystallizes at 500 °C in air; whereas, in a water vapor atmosphere, structural rearrangement is induced and crystallization occurs from 350 °C, confirmed by X-ray diffraction (XRD) characterizations. Water vapor also accelerates the particle growth rate by heating at 400 °C. These results indicate a particle size reduction and the potential for future selection of cathode materials for fabricating composite electrodes of ASSBs, and further, demonstrate a low-temperature heating process that can suppress compositional shifts and side reactions. Finally, the microstructure and Li^+^ conductivity of compacts fabricated using ultrafine LATP particles is evaluated.

## Experimental section

### Preparation of amorphous LATP powder

The starting LATP powder, with an initial median size of 15 μm, was purchased from Toshima Manufacturing Co., Ltd., Japan (99.9% purity, the atomic ratio of Li : Al : Ti : P = 1.29 : 0.30 : 1.73 : 2.99 reported by the manufacturer). The detailed powder properties of LATP are shown in Fig. S1 and Table S1.† The particle size reduction of LATP was conducted *via* wet planetary ball milling. The LATP powder (2 g) and ethanol (36 mL, >99.5% purity, Kishida Chemical Co., Ltd., Japan) were placed in a stainless-steel vessel (170 cm^3^) containing Y_2_O_3_-stabilized ZrO_2_ balls (200 g, *ϕ* 0.5 mm, Nikkato Corp., Japan). The vessel was sealed and then processed in a planetary ball mill (High-G BX254E, Kurimoto Ltd., Japan). The milling was conducted at 20 °C for 4 h under a centrifugal acceleration of 150 G by controlling the revolution speed. The ratio of rotation/revolution speeds was fixed to 0.497. After milling, the resultant slurry and balls were separated by a screen. The milled-LATP dispersion was slowly condensed while stirring on a hot plate. Finally, the residual solid (*i.e.*, amorphous LATP (denoted as a-LATP)) was fully dried overnight in an oven at 100 °C and then ground with an agate mortar.

### Crystallization of a-LATP

To study the crystallization behavior, calcination of a-LATP was conducted in air and water vapor atmospheres using a tubular furnace equipped with an evaporator. The powder sample mounted on an Al_2_O_3_ boat was placed inside the furnace. Then, the sample was heated in air at a rate of 5 °C min^−1^ to 300–500 °C and held for 1 h. Calcination in water vapor was conducted at a vapor pressure of 0.1 MPa. To establish a controlled water vapor atmosphere during calcination, pure water was pumped at a flow rate of 1 mL min^−1^ into the evaporator, which was heated above 100 °C. The generated water vapor was directly introduced into the tubular furnace without any carrier gas and allowed to follow during the holding time. The outlet of the furnace opened into the laboratory atmosphere.

### Sintering of a-LATP

The a-LATP powder was calcined at 500 °C for 1 h in air to eliminate surface adsorbed materials and then sieved to under 20 μm. The green pellets of calcined a-LATP (*ϕ* 10 mm) were prepared by uniaxial pressing at 200 MPa. The sintering was conducted at 800–950 °C for 6 h in air at a heating rate of 5 °C min^−1^. After sintering, the bulk and apparent densities of the pellets were determined by a geometric measurement and the Archimedes method, using ethanol as the displacement fluid, respectively. In this study, the theoretical density of LATP was set to 2.94 g cm^−3^.

### Material characterization

The crystalline phases of the samples were characterized by powder XRD (D2 PHASER, Bruker AXS, Germany) using Cu-Kα radiation. The diffraction patterns were collected with steps of 0.02° (2*θ*) and a counting time of 0.5 s per step. The crystallinity of the heated products was estimated from the peak areas of the crystalline phase within the amorphous pattern after subtracting the background. The specific surface area (*S*_w_) of the powder was estimated from the N_2_ adsorption measurements (3Flex, Micromeritics Ltd., USA). Prior to each measurement, the powder sample was outgassed under vacuum for 3 h at 120 °C. The *S*_w_ value was calculated using the Brunauer–Emmett–Teller (BET) method. The equivalent particle size (*d*_BET_) was calculated using the following equation: *d*_BET_ = 6/(*ρ* × *S*_w_), where *ρ* is the theoretical density. The particle morphologies of a-LATP and microstructure of the sintered pellets were examined using transmission electron microscopy (TEM; JEM-2100F, JEOL Ltd., Japan) and scanning electron microscopy (SEM; JSM-6010LA, JEOL), respectively. The solid structure, including chemical bonds and surface functional groups, was analyzed by diffuse reflectance Fourier-transform infrared spectroscopy (FTIR; IRSpirit, SHIMADZU Corp., Japan). The powder sample (1 mg) was mixed with dry KBr (100 mg, Merck KGaA, Germany) and filled into the sample holder. Raman spectroscopy (LabRAM ARAMIS, Horiba Jobin Yvon, France) was used to identify structural changes in the crystal before and after milling/crystallization. A 532 nm single-frequency laser was used as an excitation source. Simultaneous thermal and generated gas analyses of the a-LATP powder were conducted by thermogravimetric-differential thermal analysis (TG-DTA; TG-DTA8122, Rigaku Corp., Japan) equipped with a gas chromatography-mass spectrometer (GC-MS; JMS-Q1500GC, JEOL). The sample was heated up in an Al_2_O_3_ pan to 700 °C at a rate of 20 °C min^−1^ under either 20% O_2_/He or a pure He flow of 200 mL min^−1^. The mass spectra were collected by using both electron ionization (EI) at 70 eV and photoionization (PI) at 10.2 eV. For measurements of the Li^+^ conductivity, both sides of the sintered LATP pellets were polished and sputtered with Au as the ion-blocking electrodes. The effective area of Au was approximately 28 mm^2^. AC-impedance measurements were performed with an AC signal of 50 mV amplitude over a frequency range of 10 to 10^6^ Hz at 25 °C using a potentiostat/galvanostat (VMP3, BioLogic Sciences Instruments, France).

## Results and discussion

### Preparation and characterization of ultrafine a-LATP powder

**Fig. 1:**
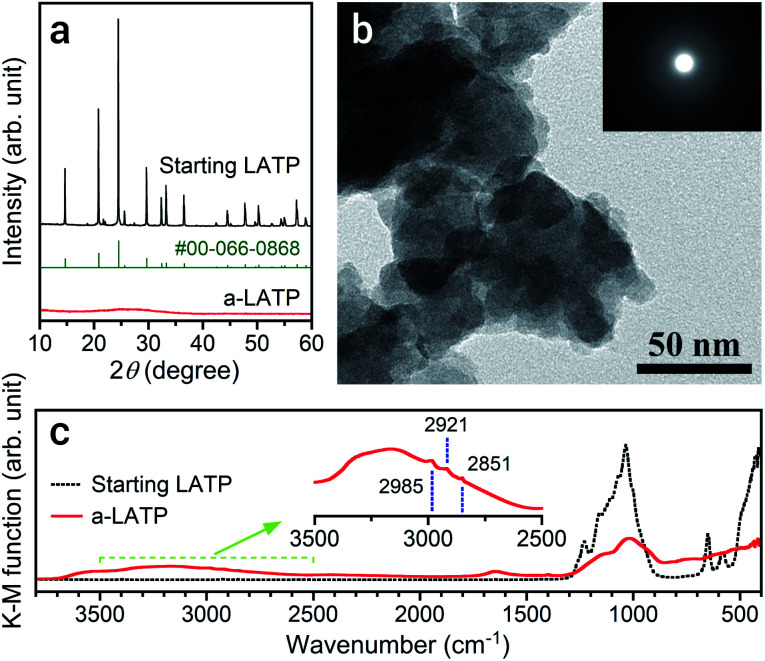
(a) XRD patterns, (b) TEM and SAED images, and (c) FTIR spectrum of the a-LATP compared with those of the starting LATP powder. The standard XRD peaks of LATP from ICDD PDF #00-066-0868 are also given in (a).

The particle size of LATP reduces with a decrease in ball size and an increase in milling time on a planetary ball milling in ethanol (Fig. S2†). The smaller balls prompt grinding at a shorter time. Milling for 4 h with balls of *ϕ* 0.5 mm produces ultrafine LATP powders with a calculated particle size of approximately 5 nm (*d*_BET_). This process succeeded a far shorter time for achieving ultrafine particles than that reported by Xu *et al.*,^21^ who conducted milling for 40 h to achieve LATP powders with sizes of 30 nm. Fig. 1 shows the powder characteristics of the resulting a-LATP powder. The crystalline phase turns into an amorphous phase after milling (Fig. 1a). The single-nanometer size of a-LATP calculated from the *S*_w_ value was confirmed by the TEM observations, exhibiting a primary particle size below 10 nm and agglomerates of several tens of nanometers (Fig. 1b). Surface-activated ultrafine particles produced by milling strongly bind to each other and agglomerate. The selected area electron diffraction (SAED) image further confirms the amorphous nature of the milled LATP (Fig. 1b, inset). The disruption of the crystal structure by high-energy milling also creates a change in the chemical bonds within LATP. The FTIR spectrum before milling exhibits defined chemical bonds in the LATP crystal structure^22,23^ (Fig. 1c): <500 cm^−1^, (PO_4_) stretching and bending bands; 500–700 cm^−1^, (TiO_6_) stretching bands; and 900–1300 cm^−1^, chemical bonds between P and O. In contrast, these LATP bands are obscured after milling, especially the characteristic (TiO_6_) stretching bands. Instead, bending and stretching bands of hydroxyl groups appear at 1500–1750 cm^−1^ and 2500–3700 cm^−1^, respectively. Further, tiny peaks between 2800–3000 cm^−1^ are observed within the stretching band of the hydroxyl groups (Fig. 1c, inset). This area is attributed to the C–H stretching bands for methyl or methylene groups. These bands imply that organic species originating from ethanol, which is the milling solvent, adsorb onto the particle surface of a-LATP.

**Fig. 2:**
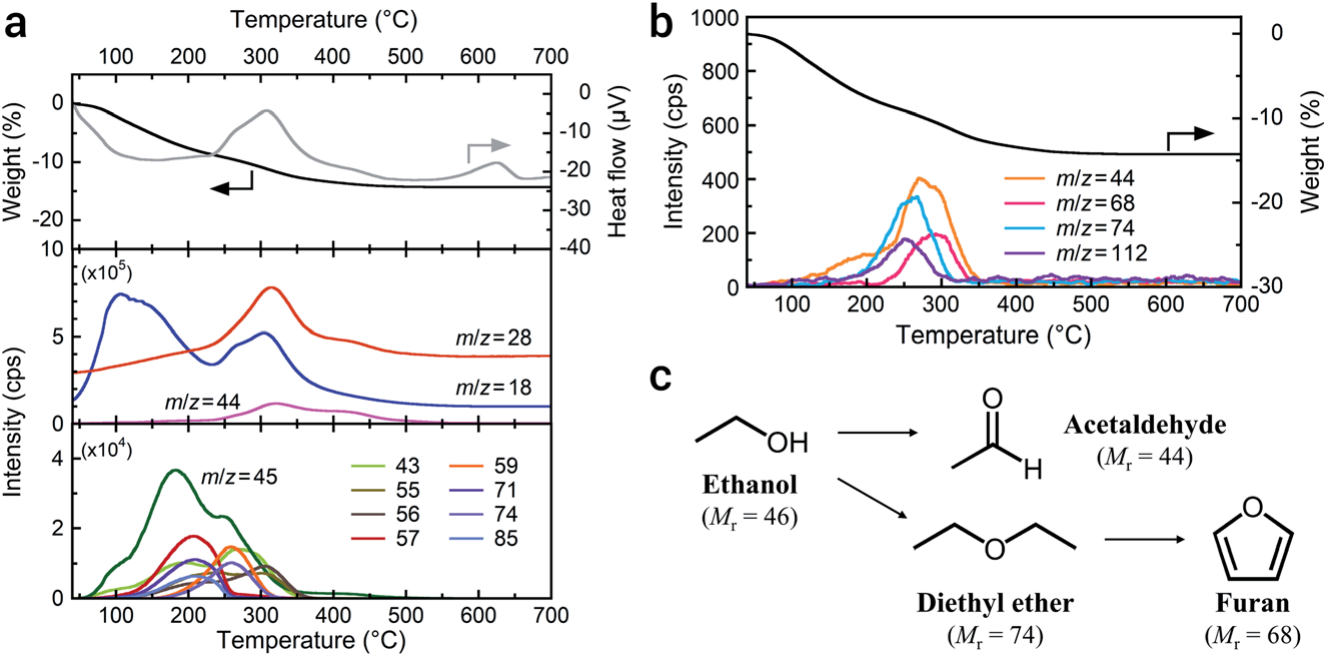
TG-DTA-MS profiles of a-LATP obtained using (a) the EI method and (b) the PI method. (c) Possible decomposition scheme of ethanol during the high-energy milling.

High-energy milling to achieve single-nanometer-sized particles potentially alters organic solvents. To determine the organic species present on the a-LATP particles, we conducted thermal analysis equipped with GC-MS. Fig. 2 shows the TG-DTA curves and mass spectra of the generated gases measured under 20% O_2_/He flow. Weight loss of a-LATP occurs up to 500 °C, decreasing by ∼14% (Fig. 2a, top). Exothermic peaks are detected at 310 °C and 620 °C, where the latter is attributed to the crystallization reaction. The mass analysis of generated gases by the EI method reveals the following findings (Fig. 2a, middle and bottom). (1) Two-step desorption of H_2_O (*m*/*z* = 18) occurs from 30 °C to 230 °C and from 230 °C to 500 °C. (2) CO_2_ (*m*/*z* = 44) is generated at 200–500 °C. (3) An *m*/*z* signal of 28, attributable to CO gas or fragmented ions of organic species, is detected at the same temperature region as the first exothermic peak. (4) A large number of signals, which are an order of magnitude smaller than the previous three signals (*i.e.*, *m*/*z* = 18, 28, and 44), occur from 50–400 °C. The starting LATP powder did not change in weight when heated up to 700 °C (Fig. S3†). Consequently, the weight loss of a-LATP up to 500 °C results from the desorption of adsorbed water and organic species. The first desorption step of H_2_O is derived from physisorbed water due to its high *S*_w_. Additional GC-MS analysis under pure He flow exhibits a typical ethanol mass spectrum,^24^ detecting fragments of *m*/*z* = 31, 45, and 46 (Fig. S4†). Therefore, ethanol and its decomposed products adsorb on a-LATP after the milling treatment.

As shown at the bottom of Fig. 2a, a large number of signals that were detected by the EI method, except for ethanol (*m*/*z* = 45), suggest the presence of multiple organic species. Alternatively, GC-MS analysis using a PI method can detect molecular mass information without fragmentation due to the low-energy ionization. The result exhibits four signals of 44, 68, 74, and 112 (Fig. 2b). These detected molecular masses indicate that the following organic species resulted from ethanol: acetaldehyde (relative molecular mass, *M*_r_ = 44), furan (*M*_r_ = 68), and diethyl ether (*M*_r_ = 74). Unfortunately, the organic species attributable to the signal of 112 are unknown. The fragment ions from 43 to 85 detected using the EI method are plausibly attributed to these compounds. Acetaldehyde and diethyl ether can be formed from ethanol through dehydrogenation and dimerization, respectively (Fig. 2c). These reactions arise on both acid and base catalysts.^25,26^ The LATP crystal has both Lewis acidic sites (metal ions) and Brönsted basic sites (PO_4_^3−^ groups and oxygen ions).^27^ Consequently, the ethanol derivatives are produced on the activated LATP surface during the high-energy milling. Although furan is potentially derived from the cyclization of diethyl ether and the subsequent dehydrogenation reaction, it is difficult to analyze side reactions of the solvent under milling.

### Water vapor-driven crystallization of a-LATP

**Fig. 3:**
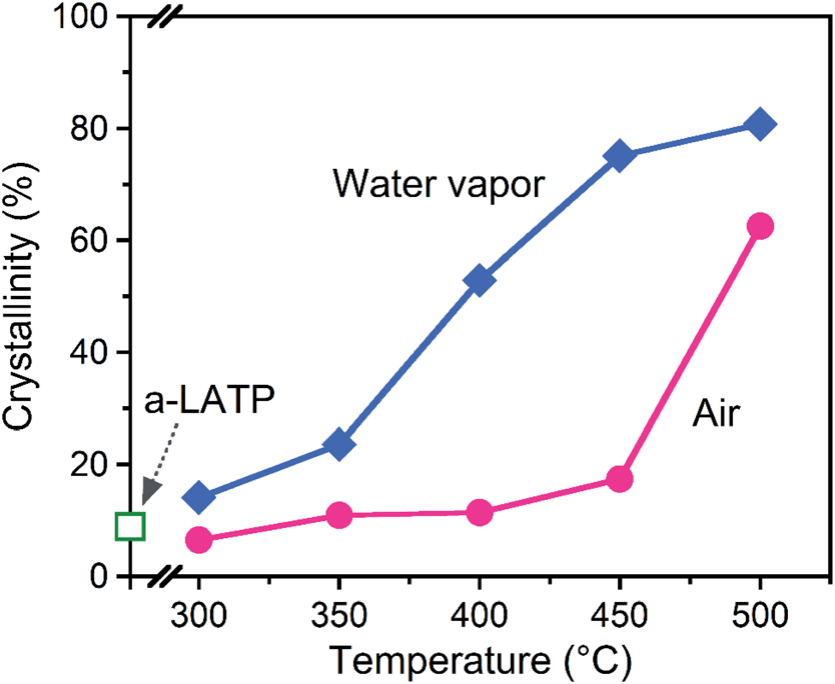
Crystallinity of the heated a-LATP products.

The crystallization behavior of a-LATP was investigated through heating from 300–500 °C for 1 h in both air and water vapor. For heating in air, XRD patterns with broad peaks are observed up to 450 °C (Fig. S5†). The diffraction peaks of LATP are clearly detected under heating at 500 °C. In contrast, these LATP peaks appear from 350 °C in water vapor. In both atmospheres, Al(PO_3_)_3_ is formed as a secondary phase. Fig. 3 plots the crystallinity of the heated products estimated from the XRD patterns. Water vapor accelerates the crystallization from low temperatures, whereas the crystallinity in air is approximately constant up to 450 °C and then increases rapidly from 500 °C. According to the above thermal analysis, a-LATP crystallizes after desorption of adsorbed water and organic species, indicating an exothermic peak at 620 °C. In the water vapor atmosphere, the crystallization is accelerated due to multiple factors, such as promoted hydrolysis of organic compounds^28,29^ and surface diffusion of hydroxyl groups.^30,31^ In particular, the increasing adsorption–desorption cycles of water vapor on metal ions under heating induce a reformation of metal–oxygen bonds by the dehydration condensation in amorphous solids.

**Fig. 4:**
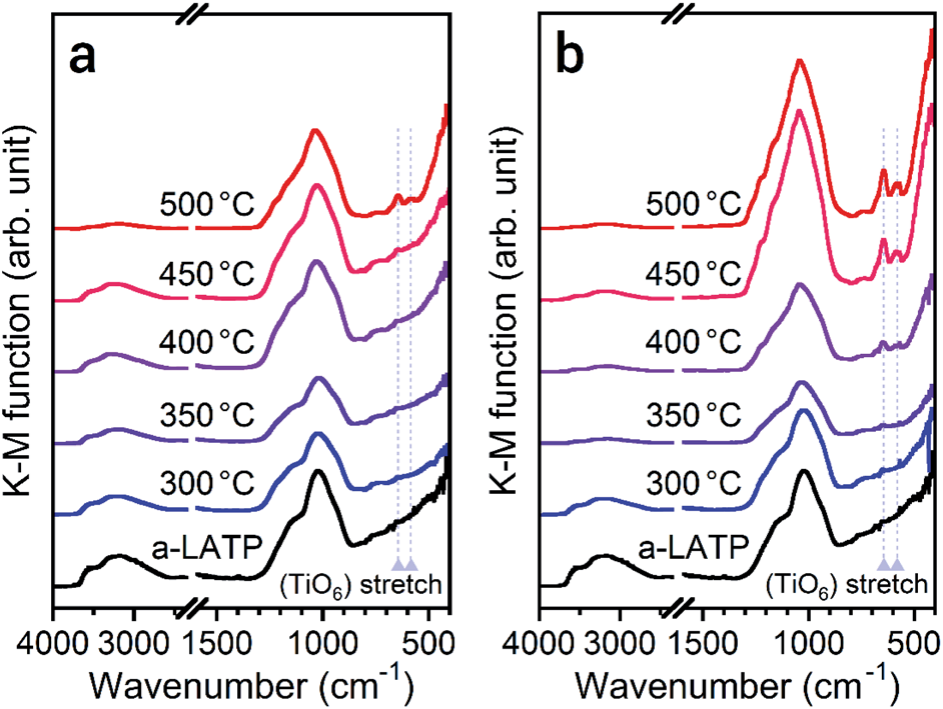
FTIR spectra of the a-LATP products heated in (a) air and (b) water vapor.

**Fig. 5:**
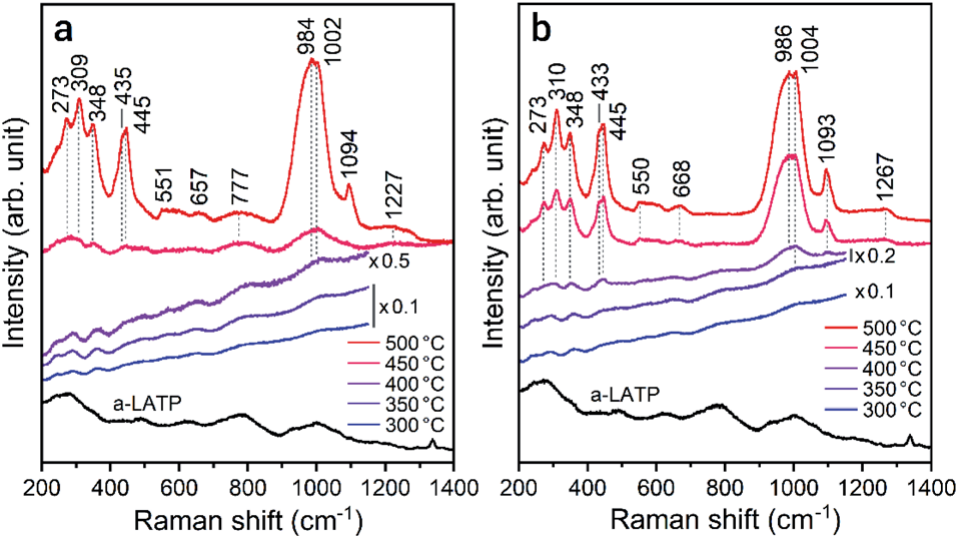
Raman spectra of the a-LATP products heated in (a) air and (b) water vapor.

To investigate the reformation of metal–oxygen bonds induced by water vapor, the bond states in the heated products were examined by FTIR and Raman spectroscopy. Fig. 4 shows the FTIR spectra of each product heated in both atmospheres. The characteristic (TiO_6_) stretching bands from 500–700 cm^−1^ reappear from 500 °C and 400 °C in air and water vapor, respectively, due to the crystallization of LATP. Although the chemical bonds between P and O at 900–1300 cm^−1^ after crystallization up to 500 °C do not show distinct band splitting as is observed in the starting powder (Fig. 1c), the intensity of these bands in water vapor is larger than that in air. The stretching band of hydroxyl groups greater than 2500 cm^−1^ remains after heating at high temperatures, especially for products in air. Fig. 5 shows the Raman spectra of the heated products. The Raman spectrum of a-LATP displays weak bands compared to the starting powder (Fig. S6†). Furthermore, these peak positions slightly differ from the crystalline phase and are relatively close to those of phosphate glasses.^32^ This is due to structural disordering caused by high-energy milling during the amorphous transformation. The humps centered at approximately 270 cm^−1^ and 780 cm^−1^ for a-LATP are identified to be the [(O–P–O) + (O–Ti–O)] and (P–O–P) chains, respectively; whereas, the hump at approximately 1000 cm^−1^ is assigned to PO_2_ symmetric stretching.^32–34^ The weak peaks at 490 cm^−1^ and 625 cm^−1^ are caused by the presence of Al^3+^.^32^ The products obtained by heating from 300–400 °C in both atmospheres suffer from the interference of the fluorescence background in the high-frequency region, which results from residual organic species.^35^ The typical Raman spectrum of the LATP phase reappears from low temperatures in water vapor. The intramolecular (PO_4_)^3−^ stretching modes appear from 900–1100 cm^−1^. The peaks at 300–350 cm^−1^ and 440 cm^−1^ are representative of the motion and the symmetrical bending mode of (PO_4_)^3−^, respectively. Moreover, the band observed at 273 cm^−1^ is assigned to the translational vibration modes of Ti^4+^.^36^ Based on these spectroscopic results, the crystal structure of LATP that was disrupted by milling is almost fully repaired by heating from 400–450 °C in water vapor but required heating to 500 °C in air.

The spectroscopic results for the crystallization behavior of a-LATP revealed that water vapor induced the rearrangement of chemical bonds and subsequent crystallization. Even after amorphization of LATP, local coordination bonds with oxygen, especially strongly covalent P–O bonds, remained. The rearrangement of the (PO_4_) tetrahedra and (TiO_6_) octahedra that build the crystal framework of LATP occurred after the desorption of chemisorbed organic species and then arose through the dehydration condensation of hydroxyl groups, which is the second desorption step of H_2_O (Fig. 2a, middle). As shown in Fig. 2a, the desorption of organic species continued up to 500 °C in air. In contrast, this desorption step occurred at lower temperatures under the water vapor environment because the fluorescence background of the Raman spectra at 350–400 °C decreased compared with that in air (Fig. 5b). Further, water molecules in the heating atmosphere assist the condensation reactions between the surface hydroxyl groups using hydrogen bonding.^37,38^ As shown in Fig. 4, the hydroxyl groups in the products heated in air remained at higher temperatures compared with those heated in water vapor. The promoted condensation reaction by water vapor triggers the rearrangement of the coordination polyhedra.

### Particle growth in water vapor

**Fig. 6:**
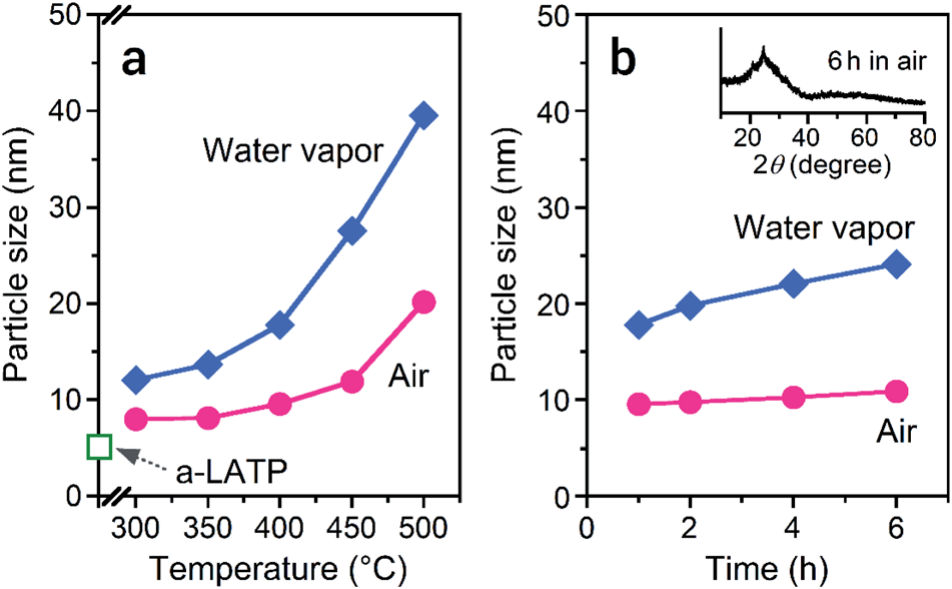
Change in particle size of a-LATP heated in air and water vapor for a (a) heating time of 1 h and (b) constant temperature of 400 °C. Inset figure shows the XRD pattern of the product after heating at 400 °C for 6 h in air.

Alongside the crystallization process, particle growth is also induced by the presence of water vapor.^39,40^Fig. 6 shows the particle size of the products after heat treatments in both atmospheres. The a-LATP particle with an initial size of 5 nm dramatically grows to 40 nm after heating at 500 °C in water vapor, but grows to 20 nm in air (Fig. 6a). Particle growth tends to occur as the crystallinity increases. The growth rate was investigated under a constant heating temperature by using a prolonged heating time at 400 °C (Fig. 6b). For heating in air, the particle size of 10 nm at 1 h is maintained at 11 nm after 6 h. In contrast, the size of the crystalline particles in water vapor gradually grows from 18 nm at 1 h to 24 nm at 6 h. Surface defects exist on the ultrafine particles; the amount of surface defects is even higher in the powders prepared by a milling method. Water molecules in the heating atmosphere adsorb on the oxygen vacancies and trigger surface diffusion of the hydroxyl groups to a neck region between the particles.^41^ The active adsorption–desorption cycles of the water molecules propagate to the particle interiors, resulting in dramatic particle growth.^42^ The heating process of a-LATP in water vapor showed the advantages of promoted rearrangement of the crystal framework, low-temperature crystallization, and control of the desired particle size. In the future, quantitative evaluation of the water vapor adsorption sites, which are the starting point for particle growth of ultrafine SE particles, will lead to the prediction of the growth rate.

### Microstructure and Li^+^ conductivity of LATP compacts fabricated using ultrafine powders

**Fig. 7:**
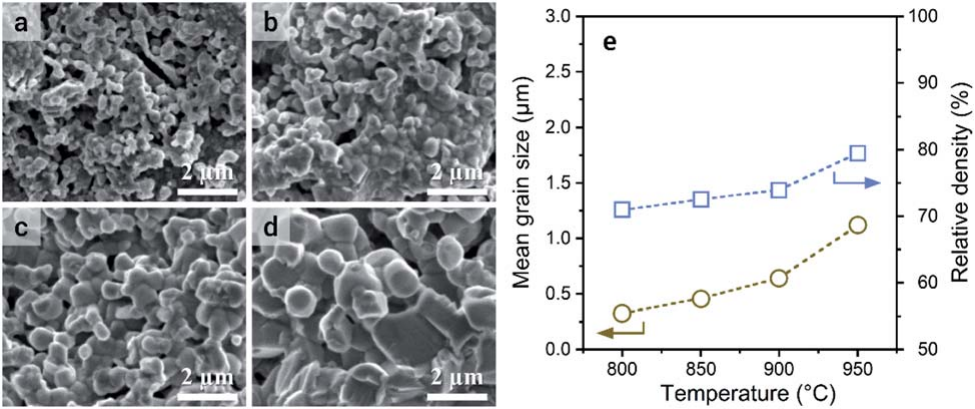
SEM images of the fracture surface of the LATP compacts sintered at (a) 800 °C, (b) 850 °C, (c) 900 °C, and (d) 950 °C. (e) Plots of mean grain size and relative density of the sintered compacts.

The sintering of a-LATP powders that were preheated at 500 °C was conducted at sintering temperatures (*T*_s_) from 800 to 950 °C in air without any additives. With increasing *T*_s_, the crystallinity recovers to the same level as that of the starting LATP powder (Fig. S7†). A small trace of a secondary phase, LiTiPO_5_, is detected up to *T*_s_ = 900 °C but is approximately diminished at 950 °C. Although the Al(PO_3_)_3_ phase was formed in the crystallization process of a-LATP (Fig. S5†), this secondary phase is eventually incorporated into LATP. Fig. 7 shows the SEM images of fracture surfaces at each *T*_s_ and the plots of grain size and density. The mean grain size was obtained by measuring at least 100 grains. Densification gradually proceeds with an increase of the *T*_s_, while the pores between grains remain. The mean grain size increases from 0.3 μm at 800 °C to 1.1 μm at 950 °C (Fig. 7e). However, the shape of grains is relatively round, even at 950 °C, as shown in Fig. 7d. In general, a change in the grain shape from round to faceted occurs with sintering progression.^43^ The sintering condition up to *T*_s_ = 950 °C for 6 h in this study falls under an intermediate stage after neck growth between the grains. This is supported by the fact that the closed pores of the sintered compacts were less than 1% from the density measurement obtained by the Archimedes method. Consequently, the internal pores are approximately open pores. The relative density, estimated from the bulk density, increases from 71% to 80%. These values are lower than those reported under similar sintering conditions.^44–46^ The reason for this insufficient densification is the agglomeration of ultrafine particles in the green pellets. As the particle size decreases, the van der Waals force acts strongly; thus, agglomerates are formed, as shown in Fig. 1b. If the agglomerates remain in the green pellets, microstructure coarsening occurs and causes pore formation (Fig. 7a and b). Furthermore, the adsorbed materials that remained even after calcination at 500 °C in air act as pore-forming agents. An improved dispersibility or surface property of ultrafine particles after milling and subsequent calcination is needed for achieving LATP electrolytes with high densities.

**Fig. 8:**
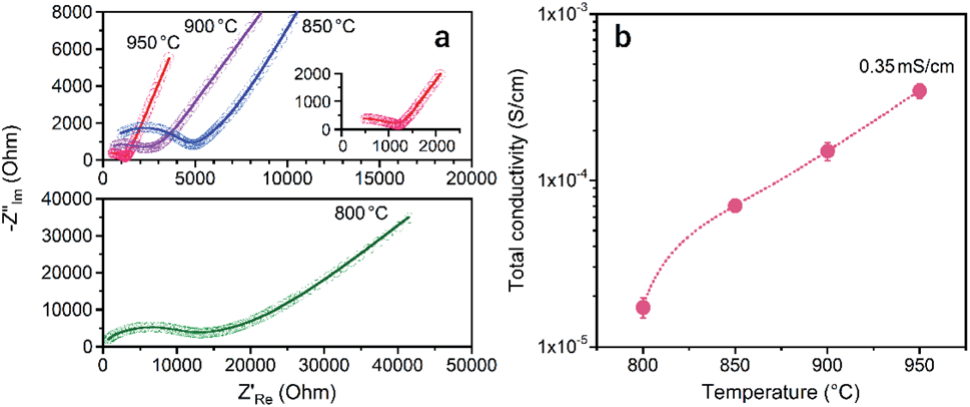
(a) Nyquist plots of the LATP compacts obtained at different sintering temperatures measured at 25 °C and (b) the total Li^+^ conductivity.

To examine the Li^+^ conductivity of the prepared LATP compacts, electrochemical tests of symmetric (Au|LATP|Au) cells were conducted at 25 °C. Fig. 8 shows the Nyquist plots of the impedance spectrum and the total conductivity (*σ*_total_) against *T*_s_. The *σ*_total_ of the LATP compacts was obtained using the following equation: *σ*_total_ = *L*/(*R*_total_ × *A*), where *L* and *A* are the thickness and effective area of the compacts, respectively, and *R*_total_ is the total resistance (for the bulk and grain boundary) calculated after fitted an optimal circuit model. As can be seen in Fig. 8b, the *σ*_total_ increases with an increase in the *T*_s_, and the LATP compacts at *T*_s_ = 900 °C and 950 °C are found to be 0.15 mS cm^−1^ and 0.35 mS cm^−1^, respectively. At lower *T*_s_ values, the values are one order of magnitude smaller (*i.e.*, *σ*_total_ = 0.02 mS cm^−1^ for *T*_s_ = 800 °C and *σ*_total_ = 0.07 mS cm^−1^ for *T*_s_ = 850 °C).

**Fig. 9:**
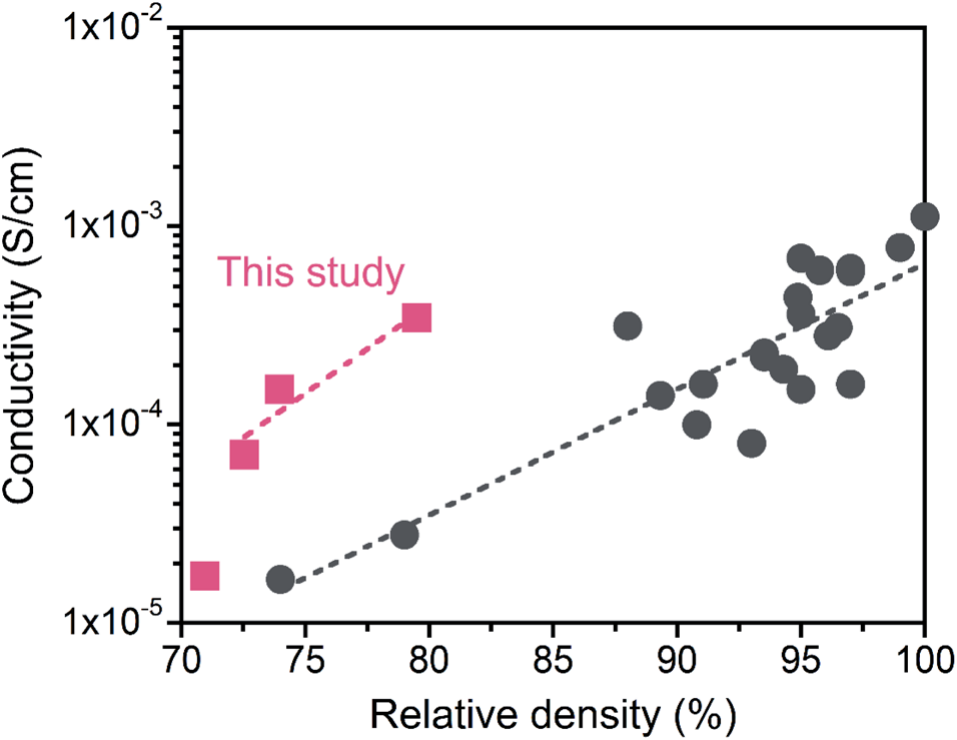
Comparison of the Li^+^ conductivity of the obtained LATP compacts with reported values as a function of the relative density. Data from ref. 45–52.

The LATP compacts obtained in this study had a good Li^+^ conductivity despite their low relative densities (below 80%). Fig. 9 plots the relationship between the Li^+^ conductivity and relative density of LATP electrolytes in the literature.^45–52^ Compared with previously reported values, the conductivity in this study exhibited high values for low relative density. The reason for the good conductivity in the “porous” LATP electrolytes is due to the fine grains. First, surface or intragranular defects with high concentrations promote the mobility of ions.^53^ Second, increasing the interfacial contact areas by using fine particles allows the expansion of the conduction path for Li^+^ transport within the grain network.^54^ However, the conductivity at the grain boundary increases with increasing relative density.^52^ For further improvement of the *σ*_total_, it is important to fabricate a dense LATP electrolyte composed of fine grains.

## Conclusions

An ultrafine (5 nm) amorphous powder of Li_1.3_Al_0.3_Ti_1.7_(PO_4_)_3_ (LATP), as a typical oxide-type SE, can be produced *via* a simple wet planetary ball milling method for 4 h in ethanol. This milling process leads to the decomposition of ethanol; thus, the altered organic species chemisorb on the amorphous LATP (a-LATP) particles, which was confirmed by FTIR and GC-MS. For the crystallization process of a-LATP, water vapor introduced into the heating atmosphere induces the rearrangement of coordination polyhedra, such as (TiO_6_) and (PO_4_), through adsorption–desorption cycles and enhances the crystallinity at a lower temperature than in air. Water vapor also contributes to the acceleration of particle growth. The preparation of ultrafine SE particles through a milling process and the control of the subsequent crystallization/particle growth steps by heating in water vapor allow for flexible adjustment of the size reduction and size ratio in the fabrication of composite electrode powders with SEs for bulk-type ASSBs. Furthermore, the LATP compacts sintered using the prepared ultrafine powders have low relative densities below 80% due to initial agglomeration. However, these “porous” LATP electrolytes exhibit a good maximum Li^+^ conductivity of 0.35 mS cm^−1^ at 25 °C. Although it is necessary to improve the dispersibility of the ultrafine particles, the fabrication of dense electrolytes composed of fine grains will provide higher Li^+^ conductivity. This study will accelerate the practical application of bulk-type ASSBs through simple nanoparticle processing.

## Author contributions

T. Kozawa: conceptualization, investigation, visualization, writing – original draft, review & editing, project administration.

## Conflicts of interest

There are no conflicts to declare.

## Supplementary Material

RA-011-D1RA02039K-s001
