# Supplementary material for: Combined wet milling and heat treatment in water vapor for producing amorphous to crystalline ultrafine Li1.3Al0.3Ti1.7(PO4)3 solid electrolyte particles
Source: RSC Adv. 2021 Apr 21;11(24):14796–804. doi: 10.1039/d1ra02039k (PMC8697808; doi:10.1039/d1ra02039k)
Supplement: RA-011-D1RA02039K-s001 [file RA-011-D1RA02039K-s001.pdf]

Electronic Supplementary Material (ESI) for RSC Advances.
This journal is © The Royal Society of Chemistry 2021

Combined wet milling and heat treatment in water vapor for producing amorphous to crystalline ultrafine $Li_{1.3}Al_{0.3}Ti_{1.7}(PO_4)_3$ solid electrolyte particles

Takahiro Kozawa*

Joining and Welding Research Institute, Osaka University, 11-1 Mihogaoka, Ibaraki, Osaka 567-0047, Japan

*E-mail: t-kozawa@jwri.osaka-u.ac.jp

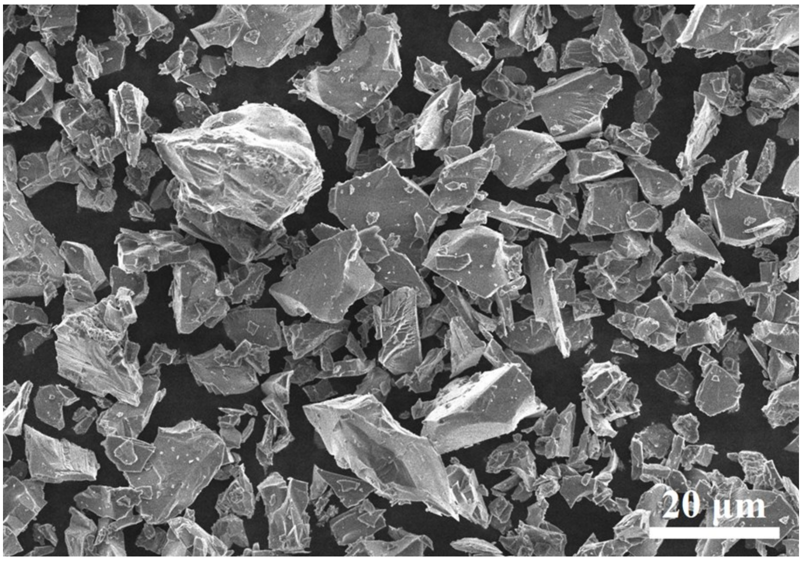

Figure S1 SEM image of the starting LATP powder.

Table S1 Impurities reported by manufacturer.

Element	Ca	Cr	Fe	Mg	Zn
mass%	0.0091	<0.0005	0.0032	0.0017	0.0005

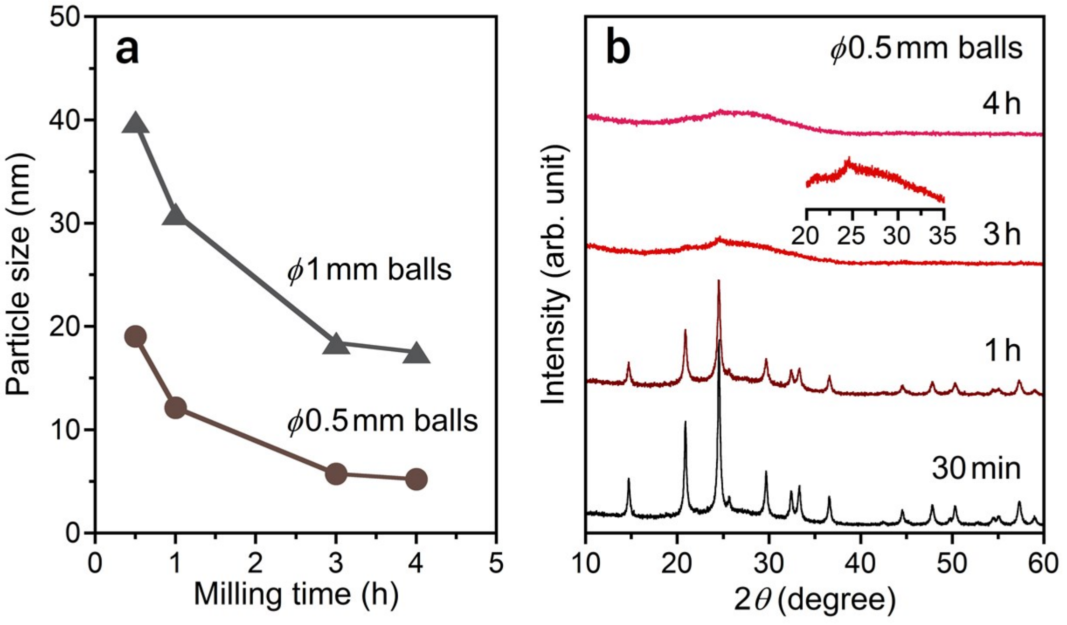

Figure S2 (a) Effect of ball size and milling time on the particle size of the milled products. (b) XRD patterns of the products obtained through wet planetary ball milling.

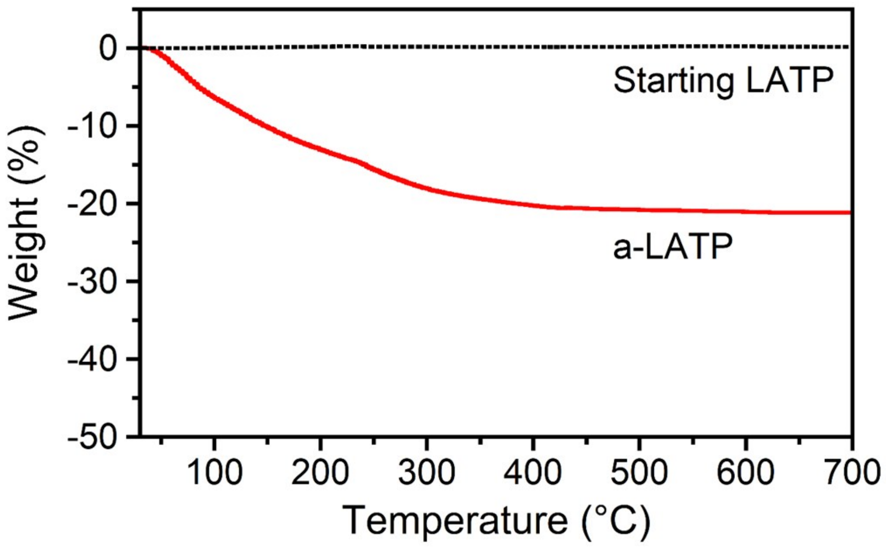

Figure S3 TG curves of the starting LATP and a-LATP powders.

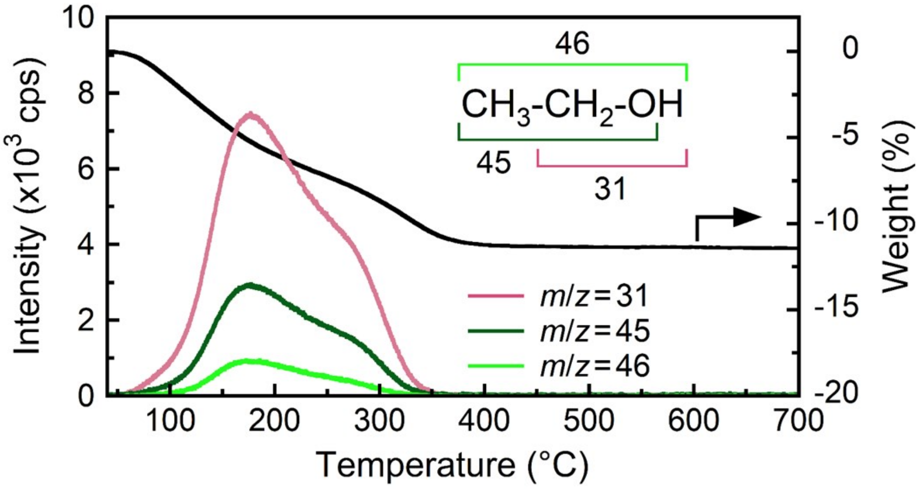

Figure S4 TG-MS profiles of a-LATP obtained using the EI method under pure He flow.

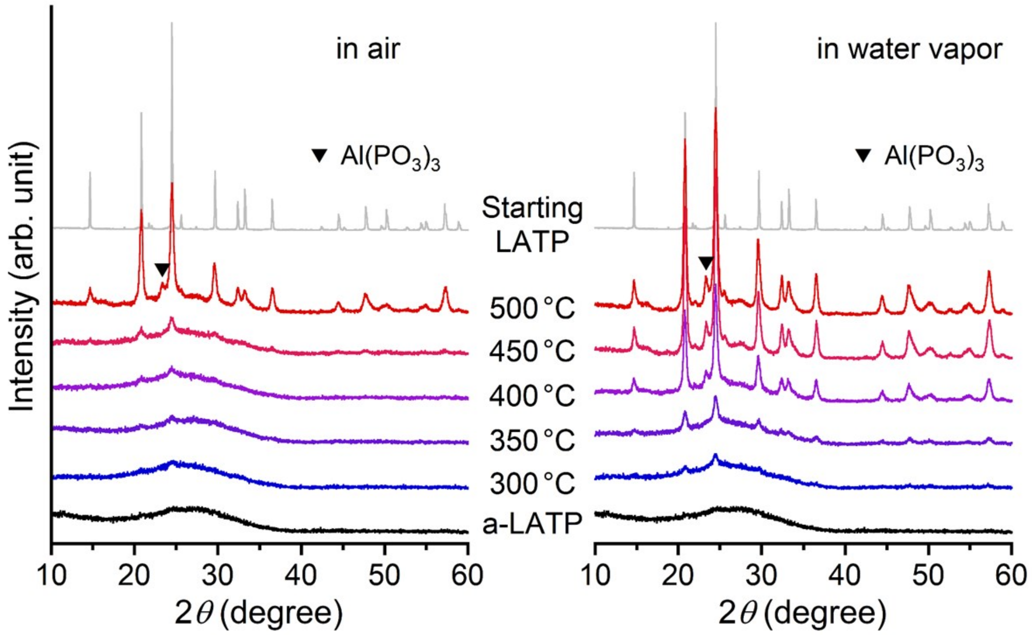

Figure S5 XRD patterns of the a-LATP products heated in air and water vapor.

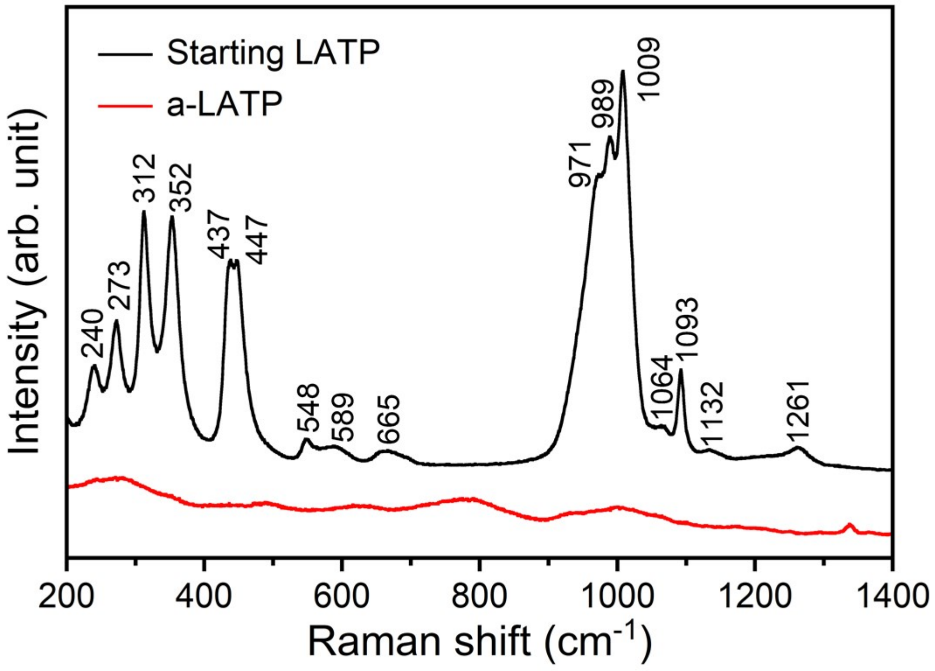

Figure S6 Raman spectra of the starting LATP and a-LATP powders.

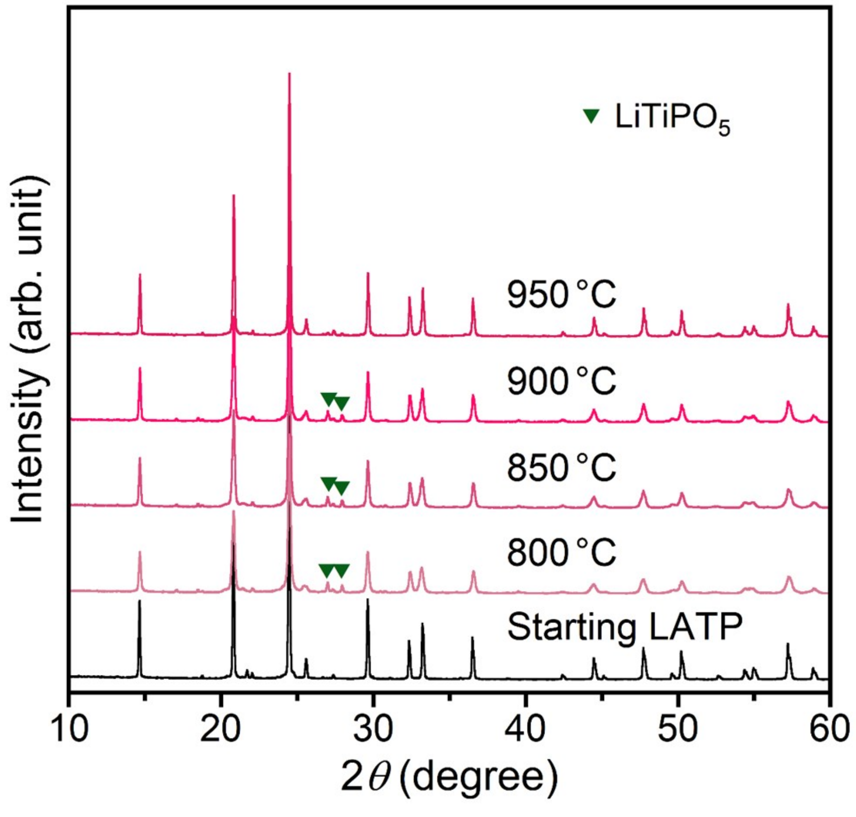

Figure S7 XRD patterns of the LATP compacts sintered at different temperatures.
